# Backyard running: Pushing the boundaries of human performance

**DOI:** 10.1002/ejsc.12190

**Published:** 2024-09-14

**Authors:** Kevin De Pauw, T. Ampe, Y. L. Arenales Arauz, X. Galloo, L. Buyse, M. Olieslagers, T. Demuyser, H. Corlùy, S. Lamarti, S. Provyn, A. M. Jones, R. Meeusen, B. Roelands

**Affiliations:** ^1^ Human Physiology and Sports Physiotherapy Research Group (MFYS) Vrije Universiteit Brussel (VUB) Brussel Belgium; ^2^ Brussels Human Robotics Research Center, BruBotics Vrije Universiteit Brussel (VUB) Brussel Belgium; ^3^ Department of Cardiology Universitair Ziekenhuis Brussel (UZ Brussel) Vrije Universiteit Brussel (VUB) Brussel Belgium; ^4^ Department of Microbiology Antwerp University Hospital (UZA) Edegem‐Antwerp Belgium; ^5^ AIMS Lab Center for Neurosciences Faculty of Medicine and Pharmacy Vrije Universiteit Brussel (VUB) Brussel Belgium; ^6^ Anatomical Research and Clinical Studies Research Group (ARCS) Vrije Universiteit Brussel (VUB) Brussel Belgium; ^7^ Department of Sport and Health Sciences University of Exeter Exeter UK; ^8^ Department of Sports, Recreation, Exercise and Sciences (SRES) University of the Western Cape Cape Town South Africa

**Keywords:** backyard running, cognitive performance, endurance performance, physiological resilience, ultra‐running

## Abstract

Ultrarunning is gaining in popularity but no information is available on the physiological and psychological responses during backyard ultrarunning events. The aim of this study was to determine changes in cognitive function, markers of physiological resilience, and running performance during a backyard‐running event. Twelve male ultrarunners (38 ± 8 years old, BMI: 23.5 ± 1.6 kg/m^2^, and VO_2max_: 60.8 ± 4.7 mL/min/kg) were monitored before, during, and after the event. Cognitive performance was determined using a cognitive test battery before, during, and after the event. During the event, the rating of perceived exertion (RPE), blood lactate concentration, and heart rate (HR) were assessed. Physical performance was investigated using the total number of completed laps and running speed per lap. Athletes completed 34 ± 17 laps equaling 227.8 ± 113.9 km with average speeds starting at 9.0 km/h and slowing down to 7.5 km/h at the end of the event. Physiological resilience (estimated using HR/speed) varied between athletes, with significantly lower values in the more proficient backyard runners at the end of the event (*p* < 0.05). HR and lactate levels remained constant, whereas a progressive increase in RPE was noticed (*p* ≤ 0.001). A significantly worsened reaction time was observed for several cognitive tasks after the event compared to baseline measures (*p* ≤ 0.05). These observations show that physiological resilience differs depending on the level of endurance performance of the athletes. Furthermore, the backyard ultrarunning event negatively impacted psychomotor speed. Therefore, the results suggest that implementing strategies that enhance physiological resilience and/or psychomotor speed could potentially have a positive effect on performance in ultraendurance activities.

## Highlights

- Backyard running events showcase varying physiological resilience, emphasizing its role in differentiating top‐performing athletes.
- Pacing dynamics and cognitive decline during ultraendurance events highlight the complex interplay between physical and mental fatigues.
- Exploring resilience and cognitive functioning during ultraendurance events is important for optimizing training and other interventions in extreme endurance challenges.

## 1 INTRODUCTION

Backyard running is an extremely demanding ultraendurance event where athletes strive to complete a 6.7 km lap every hour (https://backyardultra.com/rules/?). This distance (6.7 km) is fixed and equals a distance of 100 miles (161 km) in 24 h (https://backyardultra.com/rules/?). The extreme ultraendurance challenge is different from traditional ultramarathons in that athletes have intermittent breaks between laps. The challenge lies in finishing each lap within the hour and beginning the next lap at the start of the following hour. As a “last man standing” competition, the event concludes when the second‐to‐last participant quits, leaving the final runner to complete the ultimate lap (https://backyardultra.com/rules/?). Remarkably, several athletes have surpassed the 100‐lap mark, equivalent to an astounding 670 km (backyardultra.com/world‐rankings).

The duration of the breaks between laps varies based on lap completion time and offers a window for rest, micro‐sleep, and sustenance. These intervals also present a unique opportunity for researchers to gather data during the event, including both objective parameters, such as cognitive performance and physiological parameters, and subjective measures from questionnaires and visual analog scales. Another crucial distinction between ultramarathons and backyard running events is the significant sleep deprivation experienced by participants. Backyard running events, often spanning multiple days and nights, push the limits of endurance and mental toughness. Athletes in pursuit of current and previous world records have continuously run for durations of up to 4 days and nights. This extended wakefulness can detrimentally impact both physical performance (Thun et al., 2015) and cognitive function (Bobić et al., 2016).

In the initial stages of the event, physical fatigue predominantly triggers cessation; however, as time progresses, mental factors assume increasing importance. Elite ultraendurance athletes are renowned for their mental toughness and perseverance (Zeiger & Zeiger, 2018) in combatting the mental component of fatigue. In the context of backyard running events, athletes employ diverse strategies to counteract the adverse effects of mental fatigue on physical performance, including nutritional interventions (such as caffeine), behavioral techniques (e.g., music), and psychological approaches (e.g., extrinsic motivation) (Proost et al., 2022).

Addressing the challenges posed by often harsh environmental conditions, athletes also rely on hydration, cooling strategies, and heat acclimatization to sustain peak performance (Hermand et al., 2019). However, rigorous pursuit of human limits carries potential drawbacks, such as the emergence of visual hallucinations due to severe sleep deprivation (Hurdiel et al., 2012) and the development of overuse injuries (Scheer & Krabak, 2021). Furthermore, cognitive performance is significantly altered after an ultraendurance race (Ultra‐Trail du Mont Blanc) (Hurdiel et al., 2015), raising questions about the consequences of pushing physical and mental boundaries. Impaired cognitive performance has been associated with elevations in heart rates relative to running speed (Perrotta et al., 2022).

Running performance is affected by the maximal oxygen uptake (Noakes, 1988), running economy (Conley & Krahenbuhl, 1980), and critical velocity (Noakes, 1988), but a review of Denadai and Greco (2022) revealed that performance predictors depend on the running distance, for example, for longer distance events (5000 m, 10,000 m, marathon, and ultramarathon), blood lactate response to exercise seem to be the main predictor of performance. More recently, one pivotal parameter that emerged is durability, which can play a significant role in predicting an athlete's performance during long endurance events (Smyth et al., 2022). Durability or resilience refers to the ability of an individual to withstand functional decline following acute and/or chronic stressors (Jones, 2023). In terms of endurance exercise, resilience might, therefore, be understood to represent the ability to resist fatigue and maintain performance (Jones, 2023). One practical method to assess is through the concept of the internal‐to‐external workload ratio decoupling, which has been shown to be important in tracking changes in performance during long‐duration exercise. In running, the internal‐to‐external workload is examined via the heart rate to running speed (Jones, 2023). Notably, this metric exhibits significant interindividual variability in terms both of the magnitude and onset of decoupling, and when classified as low, moderate, and high decoupling, athletes experiencing low decoupling had better marathon performance (Smyth et al., 2022).

As the popularity of extreme ultraendurance events continues to grow, understanding the physiological and cognitive implications of participating in events, such as backyard running, is paramount for safeguarding athletes' health and promoting informed training and participation strategies. Therefore, the objective of the current study is to assess the influence of physiological resilience on backyard ultrarunning performance and to determine the impact of a backyard ultrarunning event on cognitive performance and physiological responses. We hypothesize that physiological resilience will be greater in the runners who are able to go the longest distances, and that cognitive performance will deteriorate over the course of the event independent of running distance achieved.

## 2 MATERIALS AND METHODS

Recruitment of the participants was done through mail communication. The main inclusion criterion was that the participant should participate during the “backyard ultra” held on April 15, 2023. The exclusion criterion was that participants had to be able to run a distance exceeding marathon distance. Both males and females were invited to participate, but applications were only received from males. Twelve experienced male ultrarunners participated in this study, with personal backyards records ranging from 15 to 101 laps, covering distances between 100.5 and 677 km. Participant characteristics can be found in the results section and Table 1. The backyard lap distance is set at the standard distance of 6.7 km and an elevation of 10 m. Participants started the next lap every hour or dropped out from the race. The time intervals between the laps depend on the running speed of the participant (but was usually around 5–10 min). Prior to their participation, all participants provided informed consent and the experimental protocol received approval from the local medical ethical commission of the Vrije Universiteit Brussel and University Hospital of Brussels (BUN 1432023000050).

**TABLE 1:** Performance measures of the maximal performance test.

	*V*_max_ (km/h)	Speed LT (km/h)	Speed LT2 (km/h)	La_max_ (mmol/L)	La LT (mmol/L)	La LT2 (mmol/L)	HR_max_ (BPM)	HR LT (BPM)	HR LT2 (BPM)
AV	18.6	12.3	15.9	8.9	1.5	3.9	178	141	165
SD	1.2	1.0	1.0	2.4	0.3	0.9	7	12	8
MIN	16.8	10.8	14.4	5.7	1.1	2.7	164	125	155
MAX	20.4	14.0	17.6	13.9	2.1	5.2	186	160	180

Abbreviations: AV, average; HR, heart rate; La, lactate; LT, first lactate threshold; LT2, second lactate threshold; SD, standard deviation; *V*_max_, maximal speed.

The participants were closely monitored during the “backyard ultra” event held on April 15, 2023, in Kasterlee, Belgium. At least 2 weeks before the event, each participant underwent a sports medical consultation, in which the participant was medically screened to determine whether he can conduct the preferred sports activity in a healthy way (including an anthropometric assessment, an assessment of medical history, and a thorough physical examination, including fat percentage assessment, using a skinfold caliper and Durnin & Womersley, 1974, formulas, spirometry, a resting electrocardiogram, and echocardiography), followed by a cardiopulmonary exercise test (CPET) on a treadmill (Woodway PPS series). The CPET protocol involved a starting speed of 5.4 km/h, with speed increasing by 1.8 km/h every 3 min until volitional exhaustion. During this maximal performance test, different physiological variables were measured, including blood lactate levels (Lactate Pro, Arkray), heart rate (HR, Polar), and oxygen uptake and carbon dioxide output (Metalyzer 2, Cortex). The graphing of blood lactate concentration against running speed was used to determine the first increase and second exponential increase of blood lactate concentrations representing the first lactate threshold and second lactate threshold, respectively. Running economy of the participants was calculated for every running speed of the CPET and expressed as oxygen consumption (mL) per kilogram of body weight per km.

Additionally, prior to the event, cognitive function was assessed through both a short‐term reaction time test and a longer, computerized cognitive test battery called “Cognition” (Joggle® Research, Seattle, WA, USA). Both tests were administered using iPads (Apple, CA, USA). The short‐term reaction time test began with a red screen, and as soon as it turned green, the participants were instructed to tap the screen as quickly as possible. This sequence was repeated for five times, and the entire test took approximately 12–15 s to complete. The cognitive test battery “cognition,” with an average duration of 18 min, is sensitive to multiple high‐level cognitive performance domains and has been validated with functional neuroimaging (Basner et al., 2015). It includes various tests, such as the motor praxis test (MPT; assessing sensorimotor speed), visual object learning test (evaluating spatial learning and memory), abstract matching (measuring abstraction), line orientation test (assessing spatial orientation), digit symbol substitution test (DSST; evaluating complex scanning and visual tracking), balloon analog risk test (assessing risk decision‐making), NBACK (measuring working memory), and psychomotor vigilance test (PVT; evaluating vigilant attention). To minimize learning effects, participants practiced each cognitive test before the actual assessments. Detailed descriptions of each cognitive test can be found in the work of Basner et al. (2015). The primary outcome measure of interest for each cognitive test was the average reaction time and the program also provided task efficiency scores for all cognitive tests.

During the backyard ultra event, participants were asked to perform the short‐term reaction time test every 4 laps. Additionally, the rating of perceived exertion (RPE) was recorded using the 6–20 Borg scale (Borg, 1982) and blood lactate concentration was assessed immediately after every 4 laps. Heart rate was continuously monitored throughout the event using the participants' personal HR monitor, mainly Garmin. Physical performance was evaluated based on the total number of completed laps and running speed per lap or pacing strategy throughout the event. Individual paces during the event were separated into five zones based off a five training zone model: active recovery (zone 1), long duration endurance (zone 2), extensive endurance (zone 3), intensive endurance (zone 4), and anaerobic/resistance (zone 5) (Sylta et al., 2014). For this particular ultrarunning event, we added an extra zone, that is, walking (<5 km/h).

Physiological resilience was determined using the ratio HR/running speed. To account for individual differences in backyard running performance, we analyzed average values of these variables in quartiles (0%–25%, 25%–50%, 50%–75%, and 75%–100% of the [individual] end of the event). In order to compare the running speed of the participants during the days and nights, days and nights were defined by the hour following sunset and sunrise (i.e., day 1 [10:00 a.m.–9:00 p.m.], night 1 [9:00 p.m.–7:00 a.m.], day 2 [7:00 a.m.–9:00 p.m.], and night 2 [9:00 p.m.–7:00 a.m.]).

In addition, cognitive performance using “cognition” was assessed immediately after the conclusion of the event as well as within 1 week after the event to measure cognitive recovery.

Missing data were limited to the average heart rate values of one participant due to technical problems with the sports watch and some RPE values that participants had forgotten to mention every 4 laps.

### 2.1 Statistics

All statistical analyses were performed in the RStudio version 4.2.2 (R Core Team, 2013), using the Ime4 (Bates et al., 2015), ImerTest (Kuznetsova et al., 2017), and emmeans (Searle et al., 1980) packages. The majority of the parameters exhibited a normal distribution (Shapiro–Wilk). Therefore, linear mixed‐effects regression models with or without interaction effects were applied and post hoc *t*‐tests were conducted using the Satterthwaite's method if a significant result was found in the mixed‐effect regression model. Random effects were included in the model to account for the correlation in the data due to cluster sampling (i.e., repeated‐measures). A significance level of *p* < 0.05 was employed for hypothesis testing.

## 3 RESULTS

### 3.1 Participant characteristics and physical performance

The study included participants with an average age of 38 ± 8 years old, body mass 80 ± 8 kg, height 1.85 ± 0.07 m, BMI 23.5 ± 1.6 kg/m^2^, and body fat percentage of 14.7 ± 2.3%. These participants achieved relative and absolute VO_2_max values of 60.8 ± 4.7 mL/min/kg and 4.9 ± 0.6 L/min, respectively, during the CPET. Further performance parameters of the CPET can be found in Table 1. Regarding training status of the participants, they indicated a training volume of between 76 and 100 km per week.

### 3.2 Race performance

During the backyard running event, participants completed an average of 34 ± 17 laps, covering a distance of 227.8 ± 113.9 km, with individual lap counts ranging from 14 to 63 laps, corresponding to distances between 93.8 and 422.1 km. Participants maintained an average speed between 7.5 and 9.0 km/h for each lap, with a minimum speed of 6.8 km/h and a maximum speed of 11.8 km/h. The rest periods between laps ranged between 1.1 and 25.8 min (12.4 ± 3.7 min). During the event, temperatures ranged between 8° and 18.6° and humidity between 34% and 78%.

Linear mixed effects model revealed a significant decrease in running speed in the last quartile of the backyard running event (75%–100%) compared to earlier stages (0%–25% and *p* < 0.001; 25%–50% and *p* < 0.001; and 50%–75% and *p* = 0.005). Even when accounting for HR and RPE responses, the running speed remained significantly lower at the end of the backyard running event (75%–100%) compared to the earlier stages (0%–25%, *p* < 0.001 and *p* = 0.002; 25%–50%, *p* < 0.001 and *p* = 0.010; and 50%–75%, *p* = 0.008 and *p* = 0.009, respectively). When including the effect of day and night on the running speed and taking into account the above described effect of the reduced speed during the event, results yielded a significant lower running speed during night 1 compared to day 2 (mean diff. = −0.35 km/h and *p* = 0.012). No significant differences were found between other days and nights (i.e., day 1, night 1, day 2, and night 2).

Regarding pacing strategy during the laps, participants walked (i.e., <5.0 km/h) 5.2% of their lap on average. Based on the five training zones assessed for each participant after their CPET, participants ran 85.2% in zone 1 (i.e., recovery), 9.5% in zone 2 (i.e., long duration), 0.1% in zone 3 (i.e., extensive), and 0.0% in zones 4 and 5 (i.e., intensive and resistance, respectively). No significant differences for pacing strategy during the laps were found between the higher and lower ranked participants (*p* > 0.050).

During the backyard running event, RPE significantly increased from the first quartile to the third quartile (*p* < 0.001) and from the first, second, and third to fourth quartile (*p* < 0.001) (Table 2). No significant differences were observed for HR and blood lactate measures (Table 2).

**TABLE 2:** Objective and subjective performance parameters.

Time	Running speed (km/h)		HR (BPM)		La (mmol/L)		RPE (6–20)	
	Mean	SD	Mean	SD	Mean	SD	Mean	SD
0% to 25%	8.85	0.56	126	15	1.4	0.5	9.7	2.0
25% to 50%	8.73	0.55	124	18	1.5	0.7	11.7	2.4
50% to 75%	8.53	0.39	127	19	1.6	1.4	12.5	2.5
75% to 100%	7.94	0.53	121	20	1.4	0.7	16.0	1.7

Post hoc	Mean diff	*p*‐value	Mean diff	*p*‐value	Mean diff	*p*‐value	Mean diff	*p*‐value
0–25% to 25%–50%	0.12	0.891	2	0.923	−0.1	0.998	−1.7	0.087
0–25% to 50%–75%	0.32	0.227	0	0.999	−0.2	0.850	**−2.8**	**<0.001**
0–25% to 75%–100%	**0.91**	**<0.001**	5	0.387	0	1.000	**−6.2**	**<0.001**
25–50% to 50%–75%	0.20	0.607	−2	0.897	−0.2	0.919	−1.0	0.454
25–50% to 75%–100%	**0.79**	**<0.001**	3	0.758	0	1.000	**−4.5**	**<0.001**
50–75% to 75%–100%	**0.59**	**0.005**	6	0.349	0.2	0.886	**−3.5**	**<0.001**

*Note*. Bold values are highlighting significant differences.

Abbreviations: BPM, beats per minute; mean diff, mean difference; SD, standard deviation.

### 3.3 Physiological predictors of performance

For running economy during the CPET, a significant interaction effect was found between the personal backyard record of the participant and the speed (*p* = 0.03). With an increasing speed, the running economy significantly reduced only for the best backyarders (i.e., personal record above 35 laps) (*p* = 0.03). No significant change in running economy with speed was found for participants with a personal record below 35 laps (*p* = 0.21). Furthermore, when looking at the running economy for each running speed separately, a better running economy was observed in the participants with a higher PR at 7.2 km/h (mean diff. = −33 mL/kg/km and *p* = 0.06), 9.0 km/h (mean diff. = −37 mL/kg/km and *p* = 0.01), 10.8 km/h (mean diff. = −31 mL/kg/km and *p* = 0.01), 12.6 km/h (mean diff. = −35 mL/kg/km and *p* = 0.01), and 14.4 km/h (mean diff. = −31 mL/kg/km and *p* = 0.02). At 5.4, 16.2, and 18.0 km/h, no significant effect of the performance level was found.

**FIGURE 1:**
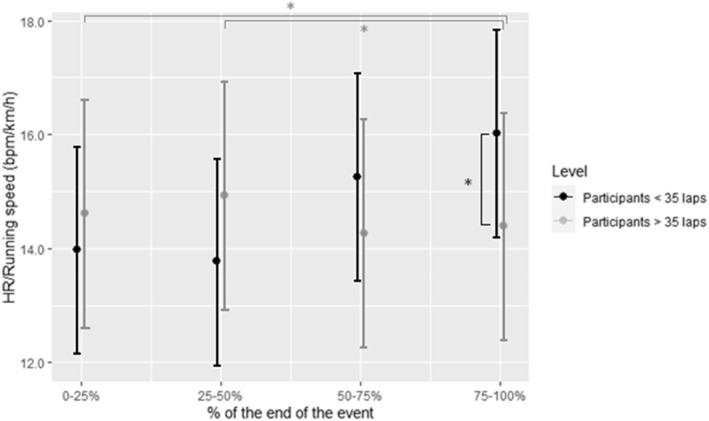
Resilience of participants running <35 and >35 laps considering HR/running speed (*p ≤ 0.028).

When examining the relationship between the HR and running speed as a measure of resilience, we observed significant interaction effects with regard to performance level (<35 or >35 laps; six participants in each group) and the last part (25%) of the backyard event. Specifically, we found that the more proficient backyard runners, that is, those who completed more than 35 laps, exhibited lower HR relative to their running speed during the final segment (25%) of the event (*p* = 0.013). There was a trend toward significance during the middle segment (50%–75%) (*p* = 0.067). In contrast, among less proficient backyard runners, that is, those who completed fewer than 35 laps, post hoc tests revealed a significantly higher HR for the same running speed in the final segment (75%–100%) when compared to the initial (0%–25%) and middle (25%–50%) segments (*p* = 0.028 and *p* = 0.016, respectively) (Figure 1). Notably, for the better performing backyard runners, there was no significant change in HR for the same running speed between the final segment (75%–100%) and the earlier segments (0%–25% and 25%–50%) (Figure 1).

### 3.4 Cognitive performance

Although the simple reaction time test did not yield significant differences (0%–25%: 292 ± 30 ms, 25%–50%: 286 ± 33 ms, 50%–75%: 289 ± 32 ms, and 75%–100%: 287 ± 28 ms), the cognitive test battery showed significantly worse reaction times during the MPT and PVT immediately after the backyard running event (EVENT) compared to baseline (pre) and recovery (post) (MPT: *p* = 0.012 and *p* = 0.022, respectively and PVT: *p* < 0.001 and *p* < 0.001, respectively) (Table 3). Additionally, worse reaction times were observed during the DSST immediately after the backyard running event compared to baseline (*p* = 0.035) with a trend toward worse reaction times compared to recovery (*p* = 0.054). No other significant differences in reaction times were observed for the other cognitive tests (Table 3). A significant main effect of the performance level was observed for the PVT test and there was a trend toward significance for the DSST test. Independent of time, the reaction time was slower for participants that performed better (i.e., >35 laps completed) during the event. For the DSST test, reaction times were 159 ms slower (*p* = 0.059) and for the PVT test 29 ms slower (*p* = 0.017). No interaction effects were observed. For VOLT, significantly higher efficiency of the tasks was observed during recovery compared to baseline (*p* = 0.018). Furthermore, significantly higher efficiency for LOT was noted at recovery compared to immediately after the event (*p* = 0.020). The DSST showed a trend toward significantly lower efficiency immediately after the event compared to baseline (*p* = 0.051). Additionally, the PVT displayed significantly lower efficiency values immediately after the event compared to baseline and recovery (*p* < 0.001).

**TABLE 3:** Reaction times and task efficiency scores extracted from the cognitive test battery.

Test	Pre		Event		Post	
	RT (ms)	Pre <‐> event (*p*)	RT (ms)	Event <‐> post (*p*)	RT (ms)	Pre <‐> post (*p*)
MPT	461 ± 51	**0.01**	509 ± 70	**0.02**	465 ± 62	0.96
DSST	926 ± 112	**0.04**	1062 ± 227	*0.05*	936 ± 156	0.98
PVT	243 ± 31	**<0.01**	283 ± 33	**<0.01**	229 ± 17	0.19

	Efficiency	Pre <‐> event (*p*)	Efficiency	Event <‐> post (*p*)	Efficiency	Pre <‐> post (*p*)
MPT	995 ± 3	0.21	992 ± 7	0.32	994 ± 3	0.95
DSST	982 ± 19	*0.05*	895 ± 160	0.12	979 ± 19	0.90
PVT	818 ± 131	**<0.01**	568 ± 211	**<0.01**	860 ± 92	0.61
VOLT	485 ± 131	*0.07*	556 ± 72	0.79	576 ± 71	**0.02**
NBACK	671 ± 159	0.97	685 ± 178	0.25	782 ± 63	0.16
AIM	555 ± 95	0.94	544 ± 127	0.10	545 ± 102	0.96
LOT	852 ± 93	0.12	794 ± 124	**0.02**	876 ± 72	0.67
BART	846 ± 188	0.93	866 ± 140	0.88	892 ± 110	0.68

*Note*. Bold values are highlighting significant differences. The *p*‐values in Italics show a trend towards a significant difference.

Abbreviations: AIM, abstract matching; BART, balloon analog risk test; DSST, digit symbol substitution test; LOT, line orientation test; MPT, motor praxis test; NBACK, *n*‐back; PVT, psychomotor vigilance test; RT, reaction time; VOLT, visual object learning test.

## 4 DISCUSSION

Ultraendurance events are gaining in popularity, prompting increased research to better understand the physiological and psychological impacts of these challenges. While there is information available on ultramarathon running, to our knowledge, this is the first study that investigated the influence of physiological resilience on running performance during one of the most extreme ultraendurance events, the backyard running event, as well as the impact of such an event on cognitive and physiological responses. This unique event stands apart from traditional ultramarathons as it involves intermittent running of 6.7 km laps and this facilitates data gathering. There were three main findings to the present study: (1) physiological resilience (as estimated using HR/running speed) was related to the level of running performance; (2) no changes in HR and lactate occurred during the event, but there was a substantial progressive increase in RPE; and (3) there was a reduced psychomotor speed after the event compared to baseline measures.

### 4.1 Running performance

Although backyard running differs from traditional ultraendurance events, we anticipated a decline in running speed throughout the event and a decline in cognitive function as the event progressed. Indeed, participants in the backyard‐running event maintained a low‐intensity running speed for an exceptionally long duration and endured significant sleep deprivation. Runners covered an average of 228 km, with one participant reaching 422 km. Notably, pacing was altered throughout the event, with lower speeds (7.5 km/h) observed toward the end, compared to the initial quarter of the event (9.0 km/h).

One main finding of the current study is the variation in physiological resilience among individuals who completed fewer than or more than 35 laps. Smyth et al. (2022) reported substantial interindividual variability in the magnitude and timing of the decoupling between the internal and external workload as reflected by the HR and running speed. This allowed the researchers to categorize athletes into low, moderate, and high decoupling groups, which proved to be a predictor of marathon performance. In the current study, we observed a similar phenomenon among less and more proficient backyard runners. Those who completed less than 35 laps exhibited significantly higher decoupling between the internal (HR) and external workload (running speed) toward the end of the event when compared to earlier phases. However, this decoupling of the internal and external workload was not observed in the case of more proficient backyard runners, those who completed at least 36 laps. Physiological resilience might represent the ability to resist fatigue and maintain performance (Jones, 2023), and this ability to withstand fatigue has been identified as a distinguishing trait of successful competitive road cyclists (Gallo et al., 2022). Consequently, it becomes apparent that physiological resilience could serve as a key factor in differentiating top‐performing athletes from the rest.

It should be noted that physiological resilience is a multifaceted trait. Other factors, such as psychological factors (e.g., mental toughness and mental fatigue) and/or changes in brain neurotransmission or oxygenation (Santos‐Concejero et al., 2015), might impact on resilience, perhaps via effects on perceived exertion (Meeusen et al., 2021). Mental toughness can be investigated using the 10‐version of the mental toughness questionnaire (Papageorgiou et al., 2019). Spragg et al. (2023) outlined that gross efficiency or running economy may also be important in resilience. Running economy/efficiency is assessed using an ergospirometric device, which we did not include during the ultraendurance event because it would have interfered with the running performance of participants. However, when determining the running economy, assessed during the CPET of all participants, we observed a significant better running economy for more proficient runners (>35 laps) compared to less proficient runners (<35 laps) at running speeds until 14.4 km/h. It is important to note that HR/running speed, which we used in the present study to reflect resilience, has some limitations including that the HR does not entirely represent VO_2_ and therefore efficiency. Specifically, while the relationship between changes in VO_2_ relative to changes in running speed will be reflected to some extent by the HR/running speed, HR is also subject to cardiovascular drift owing to environmental conditions and the possible development of dehydration during the long duration exercise. This could complicate the interpretation of the “decoupling” of the HR and running speed as an indicator of resilience.

Although cardiovascular drift occurs during each running lap, HR data obtained after each lap does not show any change throughout the event due to rest between laps, where participants could rehydrate and (partially) replenish substrate stores. Despite altered pacing (lower running speeds), there was a substantial progressive increase in RPE, particularly during the last quartile of the event compared to earlier segments. Although RPE is strongly correlated with HR and blood lactate in various populations (Scherr et al., 2013), it is also influenced by psychological factors (Potteiger et al., 2000; Winchester et al., 2012) as well as (partial) sleep deprivation (Roberts et al., 2019; Souissi et al., 2020). Indeed, this study's results align with the findings of Utter et al. (2003) who showed no significant correlation between RPE and physiological variables, such as HR and lactate, during ultrarunning races.

### 4.2 Cognitive performance

Given the significant role of psychological factors in ultraendurance events, we conducted an in‐depth investigation into cognitive functioning during and immediately after the event. While it was expected that simple reaction times would be negatively affected by the development of physical and mental fatigue during the ultraendurance event, we did not observe significant differences during the event itself. We assume that positive effects of physical exercise on motor processes in simple reaction time performance (Davranche et al., 2006) and negative effects of fatigue and sleep deprivation on simple reaction time performance (Jaffe et al., 2018; Pavelka et al., 2020) balanced out during the ultrarunning event. However, we did observe worse reaction times immediately after the event compared to baseline and recovery measurements in different cognitive tasks including the MPT, DSST, and PVT. Furthermore, the efficiency of the tasks (DSST and PVT) was adversely impacted by the ultraendurance event. These three tests activate a wide brain network, for example, the putative core brain network of the PVT comprises dorsomedial, mid‐, and ventro‐lateral PFC, anterior insula, and parietal areas as well as cerebellar vermis, thalamus, basal ganglia, and midbrain (Langner & Eickhoff, 2013). All three tasks require psychomotor speed and previous studies have demonstrated that the physical and mental fatigue reduce the psychomotor speed (Angius et al., 2022; Habay et al., 2021). The longer the duration of the ultraendurance event, the more fatigued participants become (Behrens et al., 2023). However, in the current study, it was observed that the slower reaction times did not exhibit a direct association with the longer duration of the ultraendurance event. This implies that, during an ultraendurance run, reaction times experience a noteworthy slowdown at the end of the backyard event, yet this deceleration does not intensify in proportion to the event's length, likely due to the fact that each individual went to the limit. Future research should investigate whether this phenomenon is related to the athlete's proficiency level. Additionally, investigating whether reaction times exhibit a progressive slowdown until a specific threshold of fatigue, followed by stabilization for the remaining duration of the event, would provide valuable insights.

### 4.3 Limitations

The current study adds substantial information on the impact of ultrarunning on human performance and physiology, especially physiological resilience, that might be integrated in monitoring and predicting performance during the running event. A drawback of collecting data during, such an ultraendurance event, is the possible interference with the runners' race plan, as this can impact their performance and lead to dropouts. Therefore, initially, we chose to measure a limited set of variables.

It should be mentioned that a limitation of the study relates to learning effects. We observed significantly higher efficiency in the VOLT task of the cognitive test battery “Cognition” during recovery compared to baseline, suggesting a potential learning effect. Therefore, future research should include several familiarization trials to mitigate the influence of learning before conducting experiments.

### 4.4 Future research

The duration of backyard running events can be described as a severe form of ultramarathons, often leading to (severe) sleep deprivation (Smith et al., 2023). Thus, backyard running events might also serve as a valuable model for investigating sleep deprivation, a phenomenon commonly encountered in occupations with rotating shifts and extended work hours, which is known to have a substantial impact on both mental and physical performance (Van Helder & Radomski, 1989). Given the observed increase in RPE during the backyard running event without a corresponding change in HR and lactate, further research should further explore the impact of mental fatigue. A review by Garbisu‐Hualde and Santos‐Concejero (2020) suggests that physiological, neuromuscular, biomechanical, and cognitive factors collectively limit performance during ultramarathons. Future investigations should focus on these combinations of variables, including fatigue and various blood variables, to better understand which factors alone or in combination, most accurately predict performance in ultraendurance running. In line with performance prediction (the mechanisms of) physiological resilience during ultraendurance performance should be further investigated. Additionally, long‐duration events often result in gastrointestinal distress in athletes, making it pertinent to examine how the gut microbiome composition of ultraendurance athletes is affected by such events and subsequent recovery periods. Combining gut microbiome analysis with nutritional intake can help optimize individualized nutritional strategies to reduce the risk of gastrointestinal distress. Of course, other factors, such as training status and environmental conditions also play a role in gastrointestinal distress.

## 5 CONCLUSION

Backyard running events are extreme ultraendurance challenges, pushing the boundaries of human endurance performance. Remarkably, participants in the backyard ultrarunning event completed an average of 34 laps, equivalent to over four marathons, with an initial running speed of 9.0 km/h at the event's outset, gradually decreasing to 7.5 km/h as the end approached. Physiological resilience differed depending on the level of endurance performance of the athletes, showing clear interindividual variability in the decoupling between internal and external workloads. Notably, despite the steady heart rate and blood lactate measures throughout the backyard running event, athletes experienced a progressive increase in RPE and a psychomotor speed was negatively impacted. Further research is required to better understand the psychophysiological determinants of ultraendurance performance.

## CONFLICT OF INTEREST STATEMENT

No conflict of interest to disclose.
